# The Cancer Problem: A Statistical Study

**Published:** 1915-12

**Authors:** 


**The Cancer Problem : A Statistical Study.** By C. E. GREEN, F.R.S.E. Pp. 98. Edinburgh and London : William Green & Sons. 1914. [Price 5s. net.]

For five years the writer of this book has been collecting "evidence which seemed to bear upon the problem of malignant disease, in the hope that a clue might be got to the cause and possibly the cure of the condition," and the results of this study are here embodied. The extrinsic origin of the disease is alleged to be proved by the facts that cancer is more prevalent in some districts than in others, and common in some trades and uncommon in others, and that the figures bearing on these points are fairly constant. Of possible extrinsic causes the writer argues in favour of the parasitic. Occupational incidence, local incidence and the influence of fuel are statistically considered. Finally reasons are advanced for believing that calcium preparations are inimical to cancer. We have found the views and arguments submitted to the reader in these pages to be highly interesting and suggestive.

